# P-1222. Population Pharmacokinetic/Pharmacodynamic Analysis of Cefiderocol in Chinese Patients with Complicated Urinary Tract Infections

**DOI:** 10.1093/ofid/ofaf695.1415

**Published:** 2026-01-11

**Authors:** Size Li, Shuyan Yu, Xiaojie Wu, Jinjie He, Haijing Yang, Takayuki Katsube, Ningning Xie, Honghong Dou, Juan Ye, Jing Zhang

**Affiliations:** Huashan Hospital Fudan University, Shanghai, Shanghai, China; Huashan Hospital Fudan University, Shanghai, Shanghai, China; Huashan Hospital Fudan University, Shanghai, Shanghai, China; Huashan Hospital Fudan University, Shanghai, Shanghai, China; Huashan Hospital of fudan university, Shanghai, Shanghai, China; Shionogi & Co. Ltd., Osaka, Osaka, Japan; Shionogi China Co., Ltd, Shanghai, Shanghai, China; Shionogi China Co., Ltd, Shanghai, Shanghai, China; Shionogi China Co., Ltd, Shanghai, Shanghai, China; Huashan Hospital, Fudan University, Shanghai, Shanghai, China

## Abstract

**Background:**

Cefiderocol, a siderophore cephalosporin, demonstrates potent activity against Gram-negative bacteria, including carbapenem-resistant isolates. Its standard dosing regimen (2 g every 8 h, 3 h infusion) is adjusted for renal function (creatinine clearance [CrCL] < 60 or ≥120 mL/min). However, evidence supporting adequate exposure in Chinese cUTI patients is lacking. The objective of this study was to investigate the PK/PD of cefiderocol in Chinese cUTI patients and compare differences in PK/PD versus caucasian patients with cUTI.

**Figure 1:**
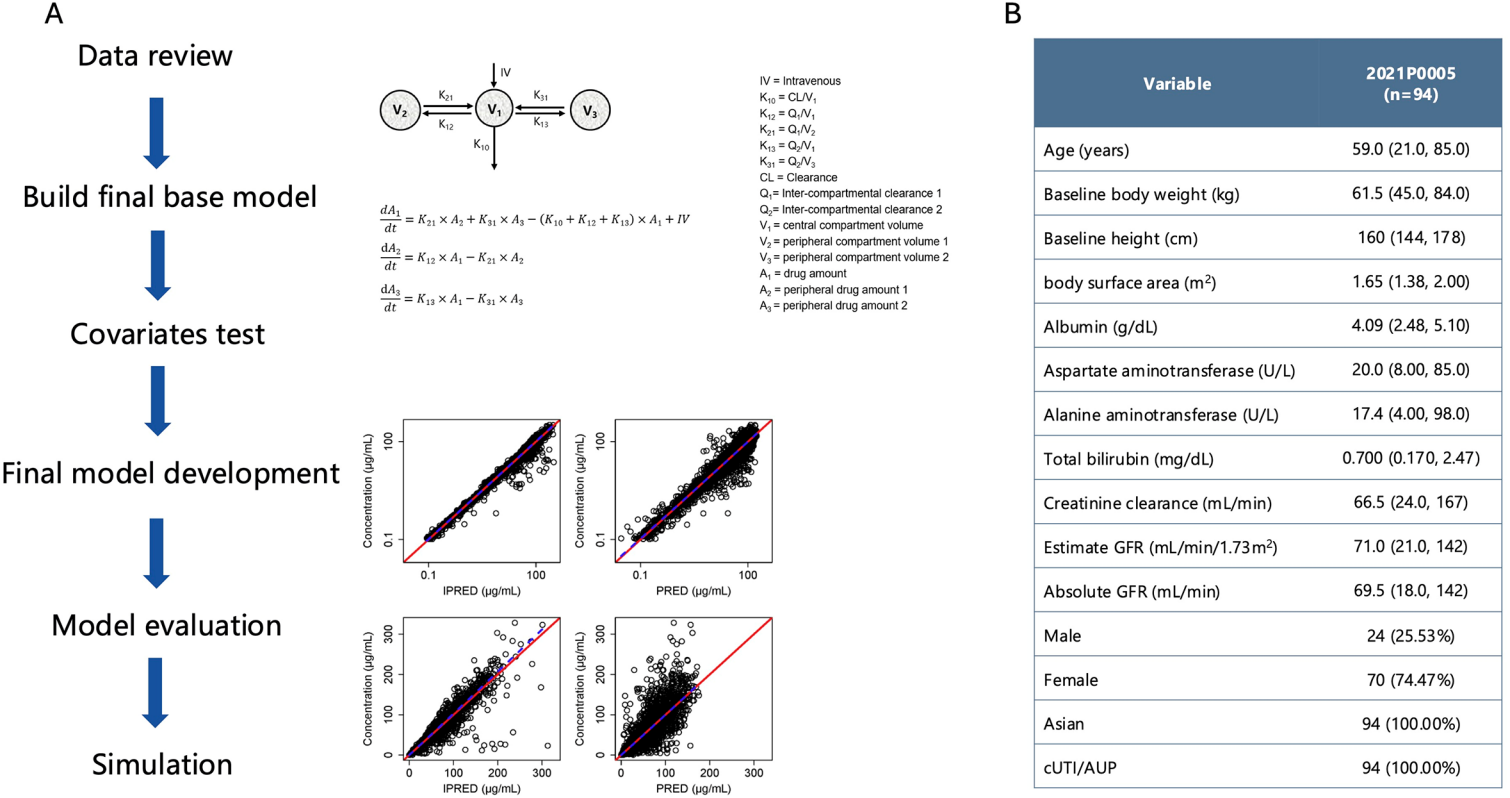
Overview of workflow of this study (A) and baseline demographic characteristic of Chinese complicated urinary tract infections patients (B). Continuous data in Figure 1B was shown as median (min, max) and categorical data was shown as number of subjects (Proportion).

**Figure 2:**
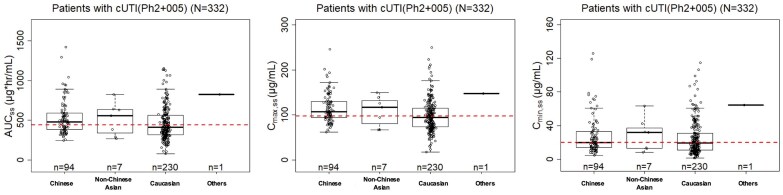
Comparison of cefiderocol exposure in Chinese cUTI patients with global cUTI patients. AUCss, area under concentration-time curve at steady state; Cmax,ss, maximum concentration at steady state; Cmin,ss, minimum concentration at steady state

**Methods:**

A published population pharmacokinetic model (3,427 plasma concentrations from 91 uninfected subjects and 425 patients with pneumonia, BSI/sepsis, or complicated urinary tract infections (cUTI)) was updated with 763 concentrations from 12 Chinese healthy subjects and 94 cUTI patients from the Phase III clinical trial in Chinese patients (2021P0005) (Figure 1). The in vitro susceptibility results of cefiderocol are derived from the 2023 surveillance of its in vitro antibacterial activity against Gram-negative bacilli isolated from clinical samples in China. The model evaluated renal-adjusted dosing, probability of target attainment (PTA), cumulative fraction of response (CFR) against pathogens, and correlations between %*f*T >MIC and clinical/microbiological outcomes.

**Figure 3:**
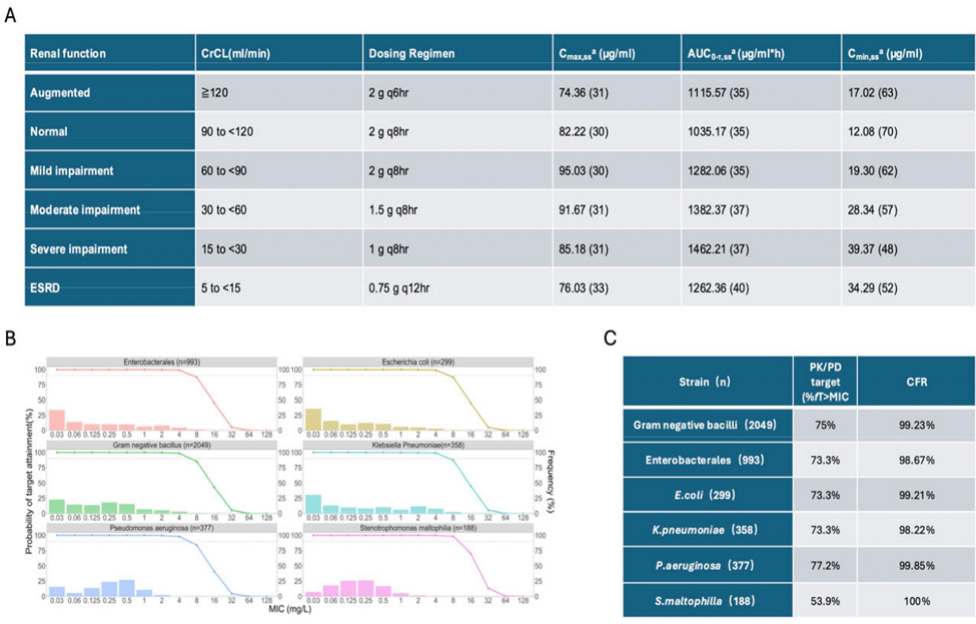
Simulated Cefiderocol exposure in Chinese cUTI patients under Renal-adjusted dosing regimens (A). Simulated probability of target attainment (B) and cumulative fraction of response (C) of cefiderocol against different gram negative pathogens. Simulation setting: n = 1000; Weight equals to 60.6 kg (CV: 30%, range from 40 to 85 kg); Albumin equals to 2.96 g/dL (CV: 30%, range from 2.62 g/dL to 3.35 g/dL); Proportion of renal function: augmented (18%), normal (46%), mild impairment (18%), and moderate impairment (18%)

**Figure 4:**
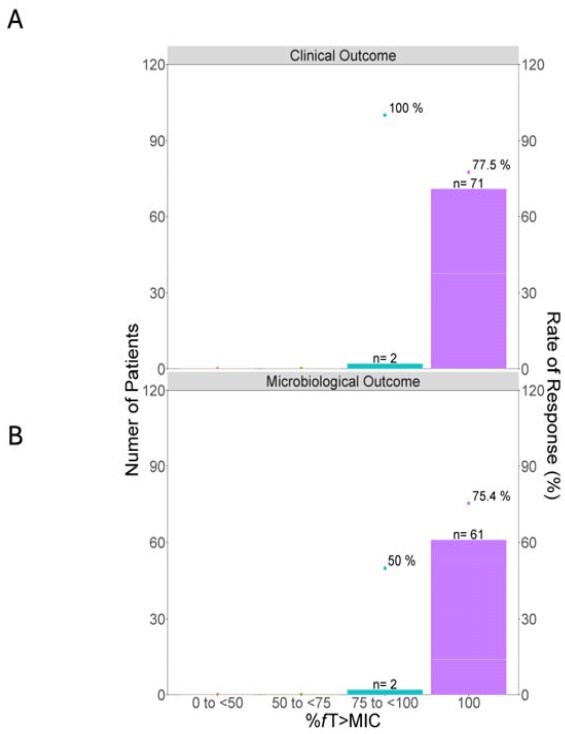
Distribution of individual %fT>MIC and individual clinical or microbiology outcome. Barplots indicate the number of patients, and the points indicate the overall response/eradication rates in this group

**Results:**

No significant exposure differences were observed between Chinese and Caucasian cUTI patients (Figure 2). Renal-adjusted dosing was validated for Chinese patients, achieving >90% PTA for MIC ≤4 mg/L and CFR >90% against *Pseudomonas aeruginosa*, *Escherichia coli*, *Klebsiella pneumoniae*, and *Stenotrophomonas maltophilia* (Figure 3). Most patients achieved 100% %*f*T >MIC, precluding quantitative exposure-response relationships (Figure 4).

**Conclusion:**

The PK/PD characteristics of cefiderocol are comparable between Chinese and Caucasian populations, with no significant differences observed. The renal-adjusted cefiderocol regimen is appropriate for Chinese cUTI patients, with expected robust microbiological efficacy.

**Disclosures:**

Takayuki Katsube, PhD, Shionogi & Co., Ltd: Grant/Research Support Ningning Xie, n/a, Shionogi & Co., Ltd: Grant/Research Support Honghong Dou, n/a, Shionogi & Co., Ltd: Grant/Research Support Juan Ye, n/a, Shionogi & Co., Ltd: Grant/Research Support

